# Drought risk in the Anthropocene: from the jaws of death to the waters of life

**DOI:** 10.1098/rsta.2022.0003

**Published:** 2022-12-12

**Authors:** James Bevan

**Affiliations:** Environment Agency, Bristol, UK

**Keywords:** climate emergency, extreme weather, environment agency, adaptation, drought, cop

## Abstract

In this closing article, Sir James Bevan, Chief Executive of the Environment Agency, sets the scene on the Anthropocene: what this new epoch means for humans and nature, how we got here, and where we need to go next. This article sets out the alarming impact that the epoch's most distinctive feature, climate change caused by human activity, is having on drought risk and extreme weather. In response to these challenges, Sir James will review the progress made by world leaders at COP26, and set out what needs to happen next to mitigate the worst impacts of runaway climate change and to adapt to impacts that are irrevocable. In particular, he will examine what needs to be done to escape what in 2019 he called the ‘jaws of death’, the point on water companies' planning charts some 20 years from now where if we don't intervene water demand will outstrip supply. Sir James will set out what the Environment Agency is doing alongside business, government, civil society and what the Royal Society can do to help. Finally this article argues why we should be optimistic we can turn the climate crisis into an opportunity to create a better world.

This article is part of the Royal Society Science+ meeting issue ‘Drought risk in the Anthropocene’.

## 1. Welcome to the Anthropocene

It is an honour to write an afterword for the world's most prestigious and historic journal, the Royal Society's *Philosophical Transactions*. So historic some would argue that when it was launched in 1665, we were in a previous epoch—the Holocene—to the one we are in now, the Anthropocene. New epochs do not come around that often. The Holocene began more than 11 000 years ago after the last glacial period and saw the dawn of human civilization. Before that the Pleistocene lasted for 2.5 million years [1]. It saw both major climate change and a massive extinction of life forms: those two facts are connected.

The Anthropocene—the epoch which started when humans first began to have a significant impact on Earth's climate, geology and ecosystems—is itself a much-contested concept. There is a live debate about when it started. Some argue that we should go as far back as 10 000 or so years to the shift from hunter-gatherers to settled farmers [2]. Others say the Anthropocene truly began about 250 years ago with the industrial revolution, as the western world's new fossil fuel-powered economy began to drive up global temperatures. And there are those who prefer to wait until the 1950s when the acceleration of fossil fuel use, deforestation, ocean acidification, urbanization, industrial-scale agriculture, habitat destruction, species extinction and wide-scale natural resource extraction made it finally incontestable that we had now significantly modified our planet [2]. But whenever the Anthropocene did start, what no-one seriously contests is that we're in it now.

## 2. Greenhouse gases and (the wrong kind of) climate change

Nor does anyone worth listening to contest the basic science of the most important feature of the Anthropocene, which is climate change caused by human activity. We know there is a natural greenhouse effect: water vapour, carbon dioxide and certain other naturally-occurring gases in our atmosphere allow sunlight to pass through the atmosphere, providing the light that we and most other life forms need; and at the same time those gases prevent the heat the sunlight brings from leaving the atmosphere, keeping the planet warm enough for life.

This process makes the Earth's temperature some 33°C warmer than it would otherwise be, which allows human life on Earth to exist [3]. Mars is inhospitable for humans because it doesn't have a big enough greenhouse effect and thus has a largely frozen surface. Venus is the opposite: it has about 150 000 times more carbon dioxide in its atmosphere than Earth, which has produced a runaway greenhouse effect and a surface temperature hot enough to melt lead.

What we are worried about is not the natural greenhouse effect, which is benign for life on Earth, but the enhanced effect caused by humans, which is the opposite. Burning fossil fuels and cutting down forests is increasing the concentration of greenhouse gases in our atmosphere. This is trapping extra heat, causing the Earth's temperature to rise and the climate to change.

We are seeing that change in the climate already. Temperatures are rising. The twenty-first century has so far been warmer overall than any of the previous three centuries [4]. The UK's top 10 warmest years since records began have all occurred since 2002 [5]. Those rising temperatures are causing rising sea levels as glaciers and the icecaps melt. And they are causing more extreme weather, including more violent, frequent and longer lasting rainfalls, droughts, fires, flooding and coastal erosion.

In England, three of the five wettest winters on record have happened in the last eight years.^1^ In the last decade, 2010 to 2019, our winters have been on average 12% wetter than they were in the three decades from 1961 to 1990 [6]. In the storms of 2020 and early 2021, water levels on many of our major rivers smashed previous records. This is why on 16 February 2021, the Environment Agency had more flood warnings in force (594) across the country than ever before [7]. Meanwhile in other parts of the world in summer 2021 we saw further violent weather, with catastrophic flooding in Germany that killed some 200 [8] people, deadly Hurricane Ida in America and devastating wildfires in Siberia, Canada, Greece and the United States.

## 3. Drought risk is rising

Climate change is also increasing drought risk, the subject of this special issue of this journal. In England, May 2020 was the driest on record [9]. The Environment Agency's estimate is that summer rainfall is expected to decrease by approximately 15% by the 2050s in England, and by up to 22% by the 2080s [10]; and that by 2100 in the southeast we will increasingly see temperatures above 35°C, and sometimes 40°C [10].

Hotter drier summers and less predictable rainfall—two effects of a changing climate—plus over-abstraction of water for industry, agriculture and the public water supply as the population grows, is a toxic combination. It means that if we don't take action, by 2050 the amount of water available in England could be reduced by up to 15%; that some rivers will have up to 80% less water on average in summer [11]; and that we will need around 3.4 billion extra litres of water a day to meet the needs of people, industry and agriculture [10]. Welcome to drought risk in the Anthropocene, UK-style.

Nature is interconnected. As climate change is causing more extremes in one part of our environment these are colliding with other effects. So drought risk brings other risks as this domino effect plays out. We see the domino effect when extreme heat causes wildfires, waste fires, soil damage and increases flash flooding due to hotter air holding more moisture. We see it in the perfect storm faced by wildlife that lives in or depends on freshwater, which is most of it: rising water temperatures, lower flows, less oxygen, deteriorating water quality are all damaging that wildlife. And thus we see how the climate emergency is also a key driver of the biodiversity crisis.

The scariest part of all of this is that we are seeing such big climate shocks today at just over 1 degree of warming above pre-industrial levels. On our present course temperature rise will soon be teetering on the edge of +1.5°C, with +2°C or more in sight, which means these shocks will intensify.

## 4. How to respond to the challenges

That is the bad news. The good news is that we still have time to avert climate catastrophe. Even better, we know exactly what we have to do to succeed—mitigate the extent of future climate change by reducing the emissions that cause it, and adapt to our changing climate so that we are resilient to its effects.

The United Nations Climate Summit, COP26, concluded in Glasgow in November last year. As the holder of the COP Presidency, the UK Government's first goal for the summit was stronger mitigation. Building on the historic commitments made at the 2015 Paris Summit, it sought to secure global net zero by 2050 and keep the 1.5 degree target within reach. That matters hugely because the effects of global warming are exponential: stabilizing at +1.5°C is much safer than +2°C, and 2 degrees is much safer than 3 degrees [12].

The second goal for COP was effective adaptation: action to shield communities and natural habitats from the effects of climate change. That means protecting and restoring ecosystems, building flood and other defences, putting warning systems in place for environmental emergencies like flood and fire, and protecting our lives and livelihoods by making our infrastructure, our agriculture and our communities more resilient. Over the two weeks of negotiations, several significant side agreements were announced: the world's two biggest emitters, the US and China pledged to boost their own climate cooperation; over 100 countries representing about 85% of the world's forests committed to stop deforestation by 2030; and more than 100 countries planned to cut 30% of current methane emissions by 2030.

The final agreement—the Glasgow Climate Pact—commits all the 197 countries involved to strengthen their efforts to mitigate the extent of climate change, adapt to its effects and finance that work. To help deliver on these promises, all countries agreed for the first time to phase-down ‘unabated’ coal power—coal-burning without a form of carbon capture. The pact also included an unprecedented goal for developed countries to double funding provided to developing countries for adaptation by 2025 [13].

Before COP26, the planet was on course for a much more dangerous 2.7°C of global warming [14]. The announcements made during the conference put us on a path to between 1.8°C [15] and 2.4°C [16]. And crucially, all countries agreed to revisit their commitments, as necessary, by the end of 2022 to put us back on track for no more than 1.5°C of warming. COP26 was never going to solve all the problems of the planet in a fortnight. But what it has done is give renewed impetus to the global effort to tackle climate change. Its success will be measured over the next several decades by whether those commitments are implemented. However successful COP26 was, it was not designed to stop climate changing or all the effects of that change, because human activity to date means that some irrevocable climate change has already happened and that more will continue to happen, even if the world stopped all carbon emissions tonight. That is why as a nation we need to be climate ready—resilient to the future hazards and potential shocks that we already know will impact on all our lives.^2^

## 5. What the environment agency is already doing to tackle the climate emergency

The Environment Agency is already playing a central role in this country's efforts to tackle the climate emergency. We regulate most of the activities—energy, industry, farming, waste management—that emit the greenhouse gases which cause climate change and are working with those industries to progressively reduce emissions. We run the new UK Emissions Trading Scheme which caps, trades and reduces emissions. We are supporting renewable and low-carbon technology in the industries we regulate. And we are trying to walk the walk ourselves through our own commitment to make the EA a net zero organization by 2030. All that is helping reduce the extent of future climate change.

We are also playing a key role in helping the country adapt to the impacts of that change. We protect people against one of its major effects, more frequent and more violent flooding, by building and maintaining the nation's flood defences, by warning and informing communities when flooding threatens, and by coming to their aid when it happens. We help design places for people to live and work that are more resilient to climate shocks, including through our role as a statutory consultee on all major developments. We create and restore habitats—wetlands, woods, marshes, peat bogs—which both help absorb carbon to reduce climate change and protect people and wildlife from its effects—drought, flood, extreme heat etc. And we are seeking to reduce drought risk by reforming our water abstraction licencing to stop people taking unsustainable amounts of water from rivers or the ground.

## 6. Escaping the jaws of death: how we are reducing drought risk in the Anthropocene

The strategic answer to how we tackle drought risk in the Anthropocene is that we tackle the climate change that is driving it. But there are also specific measures that we can take and are taking to ensure we do have plentiful water for all in future.

Enough water for all: that's something that is not talked about nearly enough. When the media and NGOs in this country talk about water their focus is almost all about water *quality*: cleaning up our rivers, lakes and bathing waters for people and wildlife. That is important, and while we have seen massive progress over the last two decades, with most of our rivers in a better state now than at any time during the Industrial Revolution, there is a lot more for all of us to do. In the last year, the Environment Agency has improved 4500 km of waterways by restoring meanders, tackling invasive species, regulating abstraction and managing major rivers for the benefit of the public, navigation and abstractors. We will continue to collaborate with businesses to help them to make the best choices for water quality, but anyone caught breaching environmental laws faces enforcement action, up to and including prosecution. We hold water companies to account to reduce pollution, tackle storm overflows and invest more of their profits into the environment; we work with farmers to support environmentally friendly farming that does not damage water quality; working with the government to develop future laws and policy that will drive better water quality—including through the Storm Overflows Taskforce; we work with non-governmental organizations and others to protect and restore chalk streams and other water environments under threat; respond to environmental incidents (one every 45 min) to stop and reverse damage to our rivers; we are carrying out a major industry-wide criminal investigation into potential non-compliance by water companies at wastewater treatment works; we prosecute the most serious polluters—53 prosecutions against water and sewerage companies since 2015 securing fines of over £138 m; and we make the case for the funding we need to monitor what is happening to our rivers and coastal waters, enforce the rules that protect them, and enhance nature rather than just slow its degradation.

But the other really big issue about water, and the one on which we should see the media and NGOs campaigning equally hard, is water *quantity*—simply having enough for people and wildlife. Good water quality is essential, but the right water quantity is existential. We need as much emphasis on the latter in the future as we have now on the former. In a speech in 2019, I talked about the jaws of death—the point on water companies' planning charts some 20 years from now when if we do not intervene, the demand for water in this country will outstrip supply and there will simply not be enough [18]. We know what to do to avoid those jaws: reduce demand, by using less water more efficiently; and improve supply, including by investing in the right infrastructure. That means we need to think strategically, radically and long term. An initiative the Environment Agency launched last year, the National Framework for Water Resources, seeks to do just that. It identifies England's long-term water needs up to 2050 and beyond, estimates how much water users in each region will need then, and which sectors (agriculture, industry, power) will use the most. Most important of all, it identifies the actions needed to ensure resilient water supplies are available to meet the needs of all users in future.

What gets measured gets done. Which is why the initiative includes important targets which the water companies have endorsed: that by 2050 they will have achieved a 1: 500 drought resilience standard (i.e. that the chances of needing severe water restrictions will be limited to no more than 0.2% in any given year); that they will get water consumption down to 110 litres of water per person per day from the current average of 150 litres or more; that they will halve leakage, which currently loses around 20% of water put into the public water supply; that they will develop new supplies through reservoirs, water reuse schemes and desalination plants; that they will move more water to where it's needed through more transfers; and that they will reduce the use of drought measures that damage the environment. We have set up mechanisms to deliver these goals and are working with the water companies, the other regulators and the government to ensure they get done.^3^

While we plan and act for the longer term, we also need to manage drought risk in the here and now. We are doing that too. The Environment Agency has a duty to safeguard water resources in England. When drought threatens we seek to reduce the impact on the environment and water users. We coordinate the efforts of the water companies, government and others to manage drought risk, at national and local level. We regulate water companies to ensure they have up to date drought plans that show how they will effectively maintain supplies without placing unnecessary burdens on the environment. We plan for drought ourselves and exercise our response to it. We seek to predict it, by monitoring the weather, surface and groundwater levels and the environment; and by analysing the effects and the prospects. We advise the government, water companies, farmers and the public on how to use water wisely. We manage the nation's use of water by regulating abstraction through permits which limit the amount people can take from the ground or our rivers and we reduce or stop abstraction when water is scarce. We help ensure the water companies have the water they need for public supply by operating our own water transfer schemes which move water between catchments. And we respond to drought incidents like fish kills to protect wildlife and re-oxygenate rivers.

## 7. What more we will do

The EA is also stepping up action to adapt to the broader climate risks the country faces. In October 2021, we published a new report on those risks and our plans for managing them [20]. It contains some sobering analysis. We judge that we will see more and worse droughts and other environmental incidents, and increasing flood risks; that the EA will not be able to protect everyone by working on its own; that climate change is making it harder to ensure clean and plentiful water we all want; that our ecosystems cannot adapt as fast as the climate is changing; and that environmental regulation designed for a status quo world is not yet ready for a changing climate. But our report also contains answers to these challenges. We think we can meet them all, as an organization and as a country, if we:

— think differently: and our thinking needs to change faster than the climate.
— collaborate better: mitigation and adaptation work best through partnerships.
— invest early in adaptive change: which pays for itself, both in terms of damage avoided and innovation unlocked.
— work with nature not against it: where we can, for example, by using trees and wetlands to reduce flood risk and absorb carbon.
— support the development of a low carbon economy.
— help businesses prepare.
— strengthen the resilience of individual communities.
— scale up all our efforts.

Our report lays out a detailed action plan for all this which we will take forward over the next 5 years.

## 8. How you can help

You can help us. As scientists, researchers, writers and problem-solvers, you can help us and the rest of the world understand better what is happening to our climate and our drought risk and what interventions will be most successful; you can help us identify new techniques to reduce carbon and enhance resilience; you can tell the world what's happening and what we need to do now to tackle it. And we and the world will listen.

The Royal Society and its members have been at the forefront of so many breakthrough moments for humanity: Newton's Principia Mathematica; Benjamin Franklin's kite experiment; Cook's journey to Tahiti to track the Transit of Venus; the first English report of inoculation against disease; Dorothy Hodgkin's confirmation of the structure of penicillin; Crick and Watson's discovery of DNA. A hundred years from now, I'd like future generations to add that another of the historic achievements of the Royal Society was the role it played in successfully ending the climate crisis.

## 9. Conclusion: reasons to be cheerful

Let me conclude on an upbeat note. I am optimistic that we will succeed in tackling the climate emergency and reducing drought risk in the Anthropocene.

First, doing so makes political sense. There is now mass public pressure around the world to solve the climate crisis. That is driving governments to take action they were not prepared to take before, such as the recent commitments from the United States on green finance and from China on coal. The UK Government is leading the way with its ambitious strategy to reach net zero.

Second, because solving the crisis makes business sense. As a water company CEO said to me recently, ‘if I don't have any water, I don't have a business’. The cost of cheap renewable energy continues to decline and the price—financial and reputational—of carbon continues to rise [21–23]. The market will help us get where we need to be.

And the third reason I'm an optimist about this is because I believe in humanity. Since the last ice age receded, we humans have done a lot of very stupid things and causing global warning that threatens to destroy us as species has to be top of that long list. But humans have also done remarkable things that have made the world we live in a far better place. Our ingenuity as a species caused this mess, and it can get us out of it.

So, let's embrace the Anthropocene for what it is: the epoch of humans. If we channel the best parts of what makes us human: not only reason, innovation and logic but also courage, empathy and a desire for justice, we can protect our planet and make it a better place for all.

## Footnotes

1 Records from 1864 – 2013/14(2nd), 2015/16(1st) and 2019/20(5th).

2 Some ideas and sentences in this section are similar to what was in Sir James' recent speech to the Association of Foreign Banks on 16 November 2021 [17].

3 All figures from this paragraph are in here [19].
